# The Management of Inflatable Penile Prosthesis Erosion and Infection following Iatrogenic Aspiration

**DOI:** 10.1155/2024/3794872

**Published:** 2024-04-20

**Authors:** Ali Baydoun, Alexander Benben, Matthew Skalak, Jordan Bilbrew, Mazen Abdelhady

**Affiliations:** Detroit Medical Center, Detroit, MI, USA

## Abstract

This case report presents a unique and previously unreported case of malfunction, infection, and erosion of an inflatable penile prosthesis (IPP) resulting from iatrogenic injury during a priapism aspiration procedure performed by an emergency medicine physician. The patient, a 75-year-old male with a history of IPP placement for erectile dysfunction, presented with urinary retention and priapism, leading to inadvertent deflation of the IPP during aspiration. Subsequent evaluation revealed a pinhole opening on the scrotum, indicating infection and erosion of the prosthesis tubing. The patient underwent emergent explantation of the infected IPP, washout, cystoscopy, and insertion of a suprapubic tube. Intraoperative cultures identified Escherichia cloacae as the causative pathogen. This case highlights the importance of thorough chart review to identify patients with IPPs before aspiration procedures and emphasizes the need for healthcare provider education regarding potential complications in this patient population. Early recognition and management of such complications are crucial for optimal patient outcomes. While IPP placement remains a highly satisfactory treatment for erectile dysfunction, this case highlights the importance of vigilance to ensure the best care for patients with penile prostheses. It is noteworthy that ultimately, a new IPP was not placed in this patient due to the patient's significant medical comorbidities.

## 1. Introduction

Since the 1980s, there have been notable improvements in the technological advancements pertaining to the design of 3-piece inflatable penile prostheses (IPPs), with a focus on enhancing concealability and optimizing surgical placement. In the last decade, notable progress has been made in the field, encompassing various enhancements such as updates to pumps, reservoirs, tubing, and cylinders [1].

The latest AUA guidelines recommend the consideration of inflatable penile prosthesis (IPP) surgery as a management option, irrespective of prior attempts at medical management [2]. In addition to postoperative glans ischemia, prosthesis infection is regarded as a highly concerning complication of inflatable penile prosthesis (IPP) surgery. This complication has been associated with significant morbidity during the perioperative period. Furthermore, prosthesis infection imposes a substantial financial strain on our healthcare system, as the documented expenses associated with its management are six times higher than the initial cost of implantation. Factors associated with an increased risk of postprosthesis placement infection include CD4 T-cell count < 300, *Staphylococcus aureus* nasal carriage, revision surgery, prior spinal cord injury, smoking tobacco, and hemoglobin A1c level > 8.5 [3]. Here, we report a case of IPP infection, erosion, and subsequent management after iatrogenic aspiration of the prosthesis.

## 2. Case Presentation

The patient is a 75-year-old male with a history of cerebral vascular accident with residual aphasia, type II diabetes mellitus, benign prostatic hypertrophy, and IPP placed in 2004 for erectile dysfunction. The patient originally presented to the emergency department for concerns of urinary retention and priapism. A 14-French urethral catheter was placed for 500 mL of clear yellow urine. With concern for priapism, an emergency medicine physician performed a dorsal penile nerve block and then placed an 18-gauge needle into the corpus cavernosum with return of clear fluid. Due to concerns for iatrogenic deflation, urology was consulted afterwards. There were no signs of active infection, and the patient was sent home with a catheter in place and instructed to follow-up with the urology clinic to undergo cystoscopy and surgical scheduling for salvage IPP removal and reinsertion. The IPP was confirmed to be deflated at the time of urologic evaluation, and the patient was sent home with a course of empiric antibiotics.

**Figure 1:**
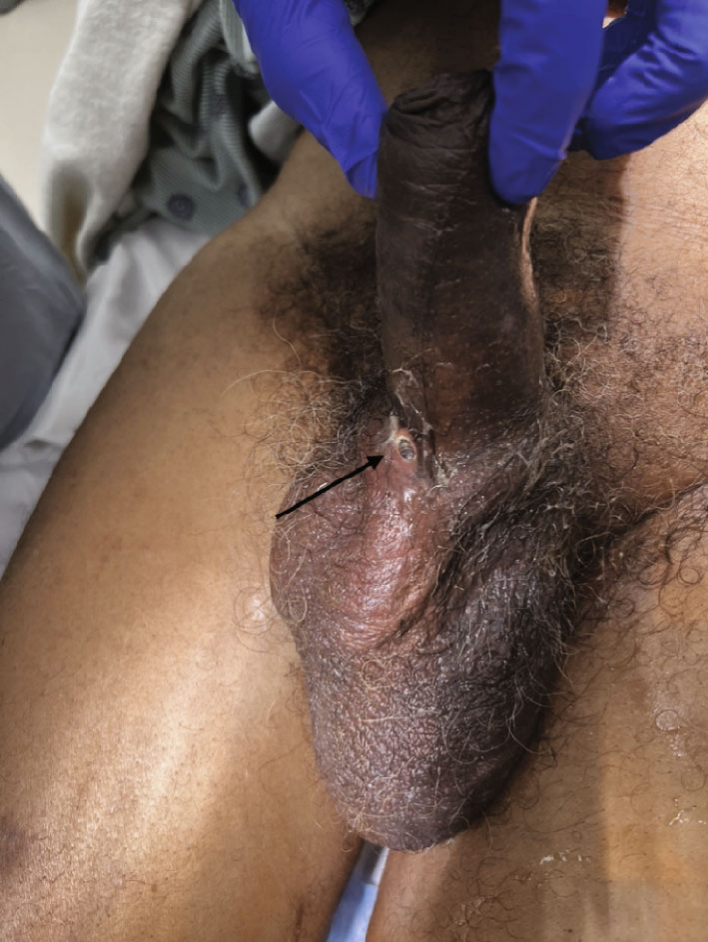
Eroded IPP tubing through the scrotal wall.

Two weeks later, he presented to the urology clinic for cystoscopy and evaluation of the prosthesis. Attempted injection of local anesthetic agent into the tip of the penis was met with resistance, and the syringe tip could not be inserted into the urethra. Of note, a urethral catheter could not be reinserted and the flexible cystoscope could not be passed. Upon further examination, a 6 mm opening could be seen on the scrotum where the prosthesis tubing could be visualized (Figure 1). There was concern for infection and erosion. The patient was transported directly to the emergency department for admission. Subsequently, he was started on broad spectrum IV antibiotics including cefepime and vancomycin.

**Figure 2:**
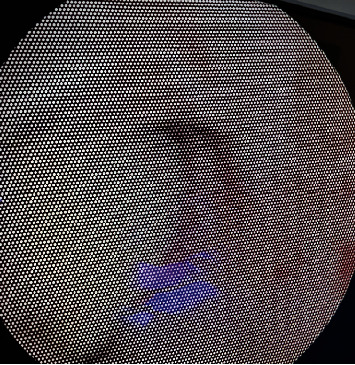
Cystoscopy showing erosion of the left corporal cylinder through the distal urethra. The cystoscope could not be passed proximally.

**Figure 3:**
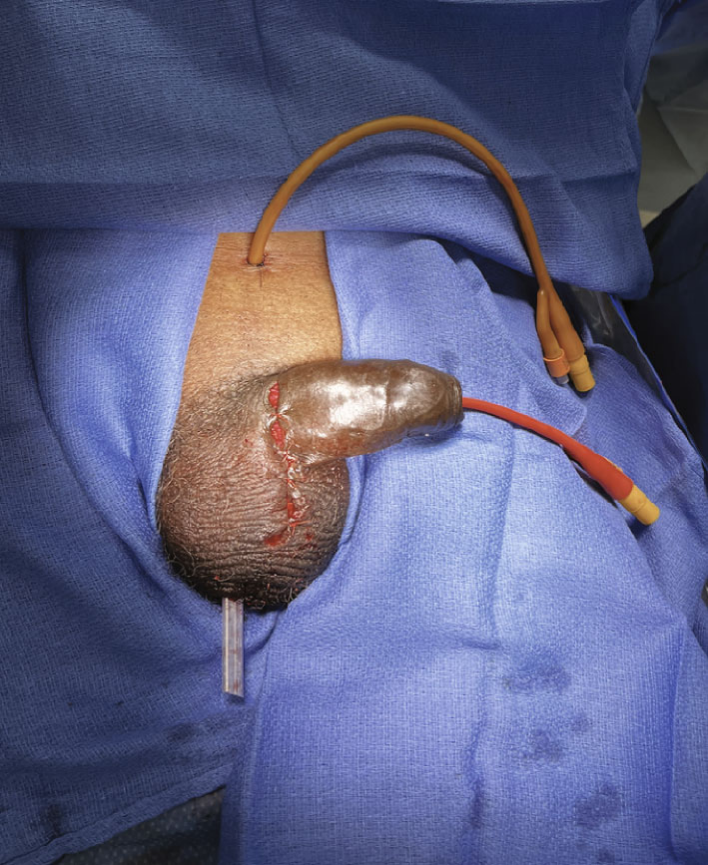
16-French suprapubic tube, 16-French council-tip urethral catheter, and Penrose drain placement.

He was taken to the operating room emergently for explanation of the infected IPP, washout, cystoscopy, and insertion of a suprapubic tube. Intraoperative flexible cystoscopy revealed the left corporal cylinder eroding through the corpora into the urethra (Figure 2). A 6 cm vertical penoscrotal incision overlying the area of erosion was made. The 3-piece IPP and all components were removed. Salvage washout was performed using hydrogen peroxide, Betadine, vancomycin, and gentamicin. Flexible cystoscopy was performed, and the true lumen was cannulated. A 16-French suprapubic tube was placed under direct visualization. A 16-French council-tip catheter was placed over a sensor wire. A quarter-inch Penrose drain was placed at the base of the scrotum for drainage (Figure 3).

Postoperatively, he was admitted into the hospital for observation. He was placed on cefepime, vancomycin, and metronidazole. Intraoperative tissue cultures grew Escherichia cloacae sensitive to Bactrim, which he was transitioned to prior to discharge. His Penrose drain was removed on postop day 5. He was discharged with the suprapubic tube to dependent drainage and the urethral catheter clamped. The patient was discharged on postop day 8. He was seen in the clinic and his urethral catheter was removed on postop day 20.

## 3. Discussion

The placement of a three-piece inflatable penile prosthesis was associated with the highest satisfaction rate among patients receiving treatment for erectile dysfunction [4].

Malfunction of penile prosthesis secondary to iatrogenic injury to the penis has rarely been reported in the literature. Dinerman and Eid report a similar injury in which there was a malfunction of a penile prosthesis after a prostatic urethral lift, which required subsequent IPP removal and replacement [5].

Regarding the management of IPP infection, the standard treatment is surgical removal of all components with extensive washout and replacement of the device at a later stage (typically 2 months). An alternative treatment option is device removal, cleansing the wound with antiseptic solution, and immediately placing a new device before closing the wound [6].

In the case report described above, the decision was made to perform explant and washout of the IPP because the patient had infection, erosion through scrotal wall, and urethral erosion. Ultimately, a new IPP was not placed in this patient due to the patient's significant medical comorbidities.

Prior to considering aspiration treatment for priapism, it is essential to perform a careful chart review, detailed history, and physical examination. O'Sullivan and Casey reported a similar case in a confused patient who underwent corporal aspiration with return of clear fluid. CT scan after aspiration revealed an indwelling penile prosthesis and artificial reservoir [7]. Correctly identifying the presence of an inflatable penile prosthesis can help decrease the incidence of iatrogenic injury and subsequent malfunction.

Furthermore, educating healthcare providers about the potential complications associated with aspiration procedures in patients with penile prostheses is imperative. This can aid in early recognition and prompt management of any issues that may arise during or after the procedure, ultimately improving patient outcomes.

## 4. Conclusion

Here, we present a unique case of malfunction and infection of an IPP due to iatrogenic injury secondary to aspiration of the penis for priapism performed by an emergency medicine physician. This case highlights the importance of careful chart review, history, and physical examination in patients with a history of IPP placement before any aspiration procedure. Identifying the presence of an IPP prior to aspiration can significantly reduce the risk of iatrogenic injury, subsequent malfunction, need for further procedures, and unnecessary healthcare costs. In this case, timely intervention, including explantation of the infected IPP, washout, and insertion of a suprapubic tube, was crucial in managing the infection and preventing further complications. It is noteworthy that ultimately, a new IPP was not placed in this patient due to the patient's significant medical comorbidities. As we continue to advance in the field of urology, ongoing research and education will be instrumental in improving the care and outcomes of patients with penile prostheses.

## Data Availability

Additional data regarding this case is not publicly available in order to protect patient anonymity.
